# YeiE Regulates Motility and Gut Colonization in Salmonella enterica Serotype Typhimurium

**DOI:** 10.1128/mBio.03680-20

**Published:** 2021-06-08

**Authors:** T. L. Westerman, M. McClelland, J. R. Elfenbein

**Affiliations:** a Department of Pathobiological Sciences, University of Wisconsin—Madison, Madison, Wisconsin, USA; b Department of Clinical Sciences, North Carolina State University, Raleigh, North Carolina, USA; c Department of Microbiology and Molecular Genetics, University of California, Irvine, Irvine, California, USA; d Food Research Institute, University of Wisconsin—Madison, Madison, Wisconsin, USA; Temple University; Carnegie Mellon University

**Keywords:** *Salmonella*, flagellar gene regulation, gastrointestinal infection, host-pathogen interactions, transcriptional regulation

## Abstract

Regulation of flagellum biosynthesis is a hierarchical process that is tightly controlled to allow for efficient tuning of flagellar expression. Flagellum-mediated motility directs Salmonella enterica serovar Typhimurium toward the epithelial surface to enhance gut colonization, but flagella are potent activators of innate immune signaling, so fine-tuning flagellar expression is necessary for immune avoidance. In this work, we evaluate the role of the LysR transcriptional regulator YeiE in regulating flagellum-mediated motility. We show that *yeiE* is necessary and sufficient for swimming motility. A *ΔyeiE* mutant is defective for gut colonization in both the calf ligated ileal loop model and the murine colitis model due to its lack of motility. Expression of flagellar class 2 and 3 but not class 1 genes is reduced in the Δ*yeiE* mutant. We linked the motility dysregulation of the Δ*yeiE* mutant to repression of the anti-FlhD_4_C_2_ factor STM1697. Together, our results indicate that YeiE promotes virulence by enhancing cell motility, thereby providing a new regulatory control point for flagellar expression in Salmonella Typhimurium.

## INTRODUCTION

Flagellum-mediated motility is a key virulence determinant facilitating gut colonization by many organisms, including nontyphoidal salmonellae (1, 2). Flagellum-mediated motility allows movement through the intestinal mucus layer toward the epithelium, allowing Salmonella to scan epithelial cells for permissive entry sites (1, 3). Upon contact with the epithelium, Salmonella invades intestinal cells, induces a neutrophilic inflammatory response, and replicates intracellularly, processes that require secretion of effector proteins through the two type 3 secretion systems (4). During early intracellular growth, Salmonella enterica serovar Typhimurium downregulates the expression of flagella to avoid immune activation induced by sensing intracellular flagellin (5). However, flagella are expressed during late intracellular infection in preparation for escape from the intracellular environment (6, 7). The dynamic expression of flagellar genes requires tight control to facilitate gut colonization while avoiding inappropriate stimulation of the innate immune response.

Flagellar biosynthesis is regulated by a transcriptional hierarchy comprised of genes in three classes. Class 1 genes encode the heterodimeric master transcriptional regulator FlhD_4_C_2_, required for expression of class 2 and 3 genes that encode the flagellar apparatus, motor force-generating elements, chemotaxis proteins, and numerous regulatory proteins (8–15). Global transcriptional regulators integrate environmental signals at the single class 1 promoter to control expression of *flhDC* (16). Small RNAs and the RNA binding protein CsrA exert posttranscriptional control of *flhDC* (17, 18). Further control of flagellar biosynthesis is mediated by regulation of FlhD_4_C_2_ function. Two proteins, each containing EAL-like domains, YdiV and STM1697 (STM14_2047), inhibit motility using different mechanisms to regulate FlhD_4_C_2_ function (19–22). YdiV binds FlhD to prevent FlhD_4_C_2_ from binding DNA and targets it for proteolysis (21, 23), whereas STM1697 restricts FlhD_4_C_2_ from recruiting σ^70^ RNA polymerase to promoters of class 2 flagellar genes (20). Together, the extensive transcriptional, posttranscriptional, and functional control of the flagellar master regulator enables tight control over the initiation of flagellar biosynthesis.

In prior work, we found a motility defect for a Salmonella Typhimurium mutant in the putative LysR-type transcriptional regulator (LTTR) *yeiE* (*STM2201/STM14_2717*) (24). LTTRs comprise the largest family of transcriptional regulators among prokaryotes and can regulate local or global gene expression in response to small-molecule ligands (25, 26). There are at least 44 annotated LTTRs encoded in the Salmonella Typhimurium genome (27–29). Although YeiE is poorly characterized in *S.* Typhimurium, it is highly conserved across related organisms. In Escherichia coli, there are no genes with clear roles that regulate flagellar biosynthesis in the YeiE regulon and no motility defect identified for a Δ*yeiE* mutant (30, 31). However, YeiE homologs improve the *in vivo* fitness of other bacterial pathogens, including Cronobacter sakazakii (*gpESA_01081*), Pseudomonas aeruginosa (*PA3398/finR*), and Vibrio cholerae (*VC2324/tehR*) (32–34). These data indicate an important role for YeiE in the pathogenesis of numerous organisms.

Despite extensive study of the regulation of flagellar biogenesis, the role of YeiE in flagellar regulation has not previously been described. The purpose of this study was to determine the role of *yeiE* in the regulation of *S.* Typhimurium flagellum-mediated motility and gastrointestinal colonization. We hypothesized that *yeiE* impacts flagellar gene expression and that dysregulated motility results in reduced gut colonization in animal models. Our work demonstrates the critical role of *yeiE* in the complex pathway regulating flagellum-mediated motility in Salmonella Typhimurium.

## RESULTS

### *yeiE* is required for swimming motility.

**FIG 1:**
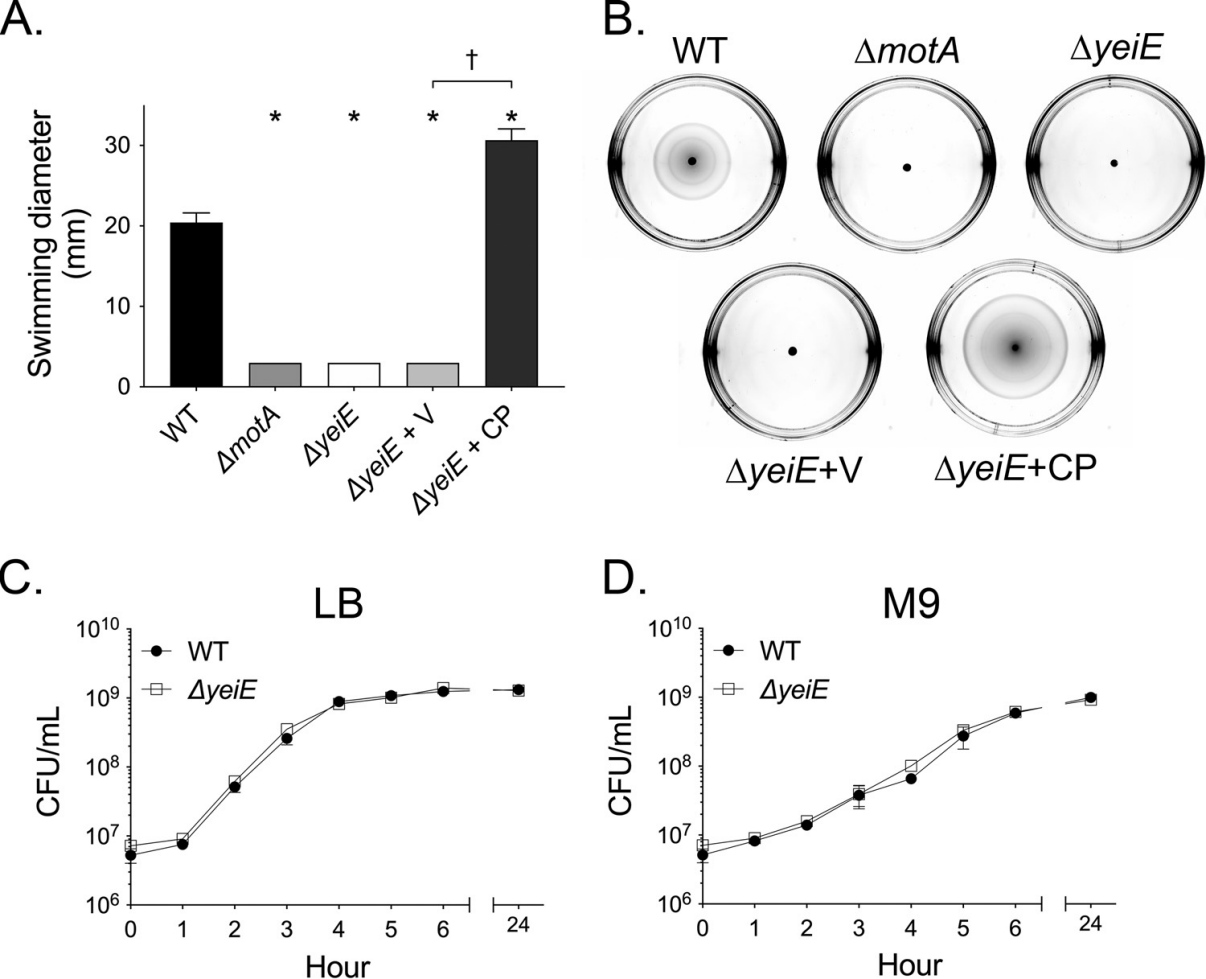
*yeiE* is required for swimming motility. (A) Normalized overnight cultures of the WT (HA420) and the *ΔmotA* mutant (JE1202), *ΔyeiE* mutant (JE973), *ΔyeiE* mutant with an empty vector (Δ*yeiE* + V; JE1511), and the Δ*yeiE* mutant with a complementing plasmid (Δ*yeiE* + CP; JE1513) were spotted onto swimming plates. Diameters of cell spread were measured 4 h postinoculation. Each assay was performed in 3 replicates on 3 independent occasions. Bars represent means ± standard errors of the means (SEM). Statistical significance was determined by Student's *t* test (*P* of <0.05). * indicates a significant difference between the WT and mutant, and † indicates a significant difference between the indicated mutants. (B) Representative photographs of swimming plates 5 h postinoculation from one experiment. Growth curves of the WT (HA420) and the Δ*yeiE* mutant (JE973) in rich (C) and minimal (D) media.

Flagellum-mediated motility is a potent agonist of the neutrophil respiratory burst (35). In a screen of Salmonella Typhimurium mutants, we found that a Δ*yeiE* mutant elicited a decreased neutrophil respiratory burst due to its lack of motility (24). To definitively link the observed motility defect to the disruption of *yeiE*, we assessed the swimming motility of the complemented Δ*yeiE* mutant. Complementation in *trans* restored the swimming motility of the *ΔyeiE* mutant to levels greater than that of the wild-type (WT) organism (Fig. 1A and B). The Δ*yeiE* mutant grew normally in both rich and minimal media (Fig. 1C and D), ruling out any potential effects of abnormal growth on swimming motility. These data demonstrate that *yeiE* plays an important role in *S.* Typhimurium swimming motility.

### *yeiE* is required for gastrointestinal colonization.

**FIG 2:**
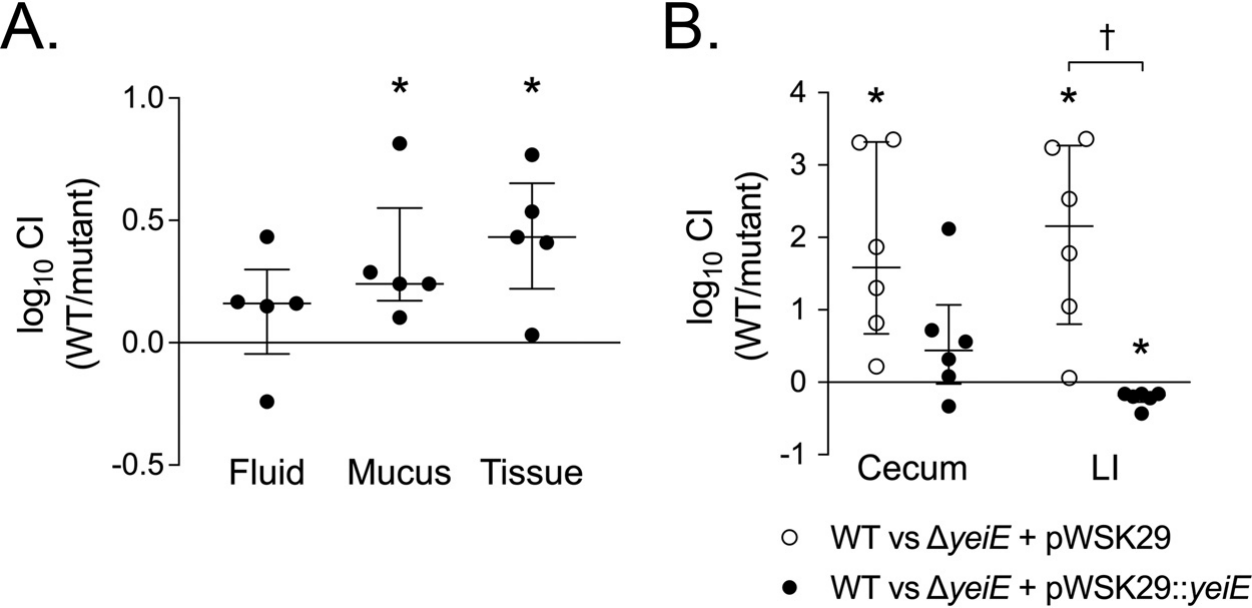
*yeiE* is required for efficient gut colonization. (A) Ligated ileal loops from five 3- to 6-week-old calves were inoculated with ∼10^9^ CFU of an equivalent mixture of the WT (HA420) and *ΔyeiE* mutant (JE973). Loops were harvested 12 h after inoculation, and luminal fluid, mucus, and intestinal tissue were processed for enumeration of CFU. (B) Six C57BL/6J mice were treated with streptomycin prior to infection with ∼10^8^ CFU of an equivalent mixture of the WT and Δ*yeiE* mutant bearing the empty plasmid (JE1511) or the complemented mutant (JE1513) by gavage. Mice were euthanized 72 h after infection and the cecum and large intestine (LI) processed for enumeration of CFU. The competitive index (CI) was determined by dividing the ratio of WT cells to mutant cells in each tissue compartment by the ratio in the inoculum. Each data point represents a single animal, with median and interquartile ranges indicated. Statistical significance was determined by Student's *t* test (*P* < 0.05). * indicates a significant difference in competitive indexes, and † indicates a significant difference between groups.

Since flagellum-mediated motility is required for efficient colonization of the gut, we hypothesized that the *ΔyeiE* mutant would have a colonization defect in animal models of enteric salmonellosis. We used the calf ligated ileal loop model of infection because calves are natural hosts for Salmonella, infections occur in the presence of an intact microbiota, and pathology is consistent with that of enteric salmonellosis in humans (36). In competitive infections with the virulent WT organism, we found that the Δ*yeiE* mutant was defective for colonization of the intestinal mucus layer and tissue but not for survival within luminal fluid (Fig. 2A). The lack of a defect in the fluid compartment suggests no effect of the Δ*yeiE* mutation on fitness in the inflamed gut, and the failure of the Δ*yeiE* mutant to penetrate the mucus layer and colonize intestinal tissue is consistent with a defect in flagellum-mediated motility.

Next, we used the murine colitis model to investigate the mechanism of the Δ*yeiE* mutant gut colonization defect. In the murine colitis model, antibiotic treatment disrupts the gut microbiota prior to Salmonella infection, allowing development of a neutrophilic typhlitis (37). As with our findings from the calf model, the *ΔyeiE* mutant had a colonization defect in the murine cecum and large intestine (Fig. 2B). The colonization defect was reversed by complementation in *trans* in both the large intestine and the cecum (Fig. 2B), definitively linking *yeiE* to the observed gut colonization defect.

**FIG 3:**
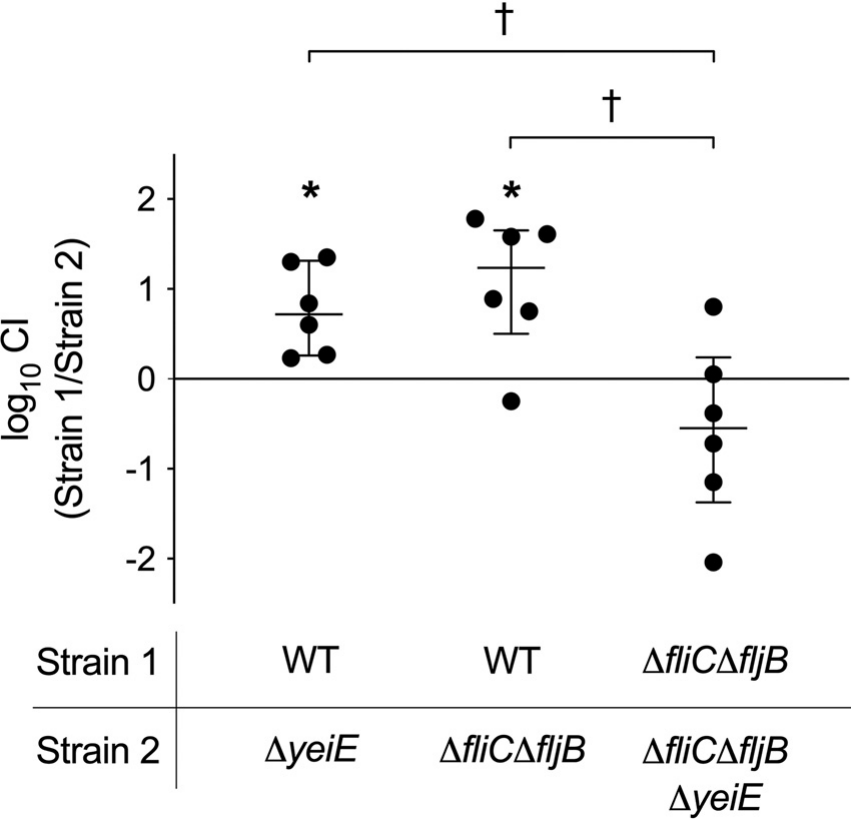
The Δ*yeiE* mutant gut colonization defect is linked to defective flagellar motility. The CI between the indicated strains in cecum from the murine colitis model was determined as described in the legend for Fig. 2. Statistical significance was determined by Student's *t* test (*P* < 0.05). * indicates a significant difference in competitive indexes, and † indicates a significant difference between groups.

We hypothesized that the motility defect of the Δ*yeiE* mutant was the likely cause of the observed gut colonization defect. To investigate the role flagellum-mediated motility played in the Δ*yeiE* gut colonization defect, we compared the competitive index (CI) of the aflagellated *ΔfliC ΔfljB* mutant with that of the Δ*yeiE* mutant. We found that the *ΔyeiE* mutant and the *ΔfliC ΔfljB* mutant had similar colonization defects in competition with the WT organism (Fig. 3). To establish whether abnormal motility was the reason for the fitness defect of the Δ*yeiE* mutant, we performed a competitive infection experiment with the *ΔfliC ΔfljB* mutant and a *ΔfliC ΔfljB ΔyeiE* mutant. We found no significant alteration in fitness of the *ΔfliC ΔfljB ΔyeiE* mutant from that of the *ΔfliC ΔfljB* mutant (Fig. 3). Together, these data demonstrate that the Δ*yeiE* mutant poorly colonizes the mammalian intestine, likely as a result of defective flagellum-mediated motility.

### Role of YeiE in flagellar regulation.

**FIG 4:**
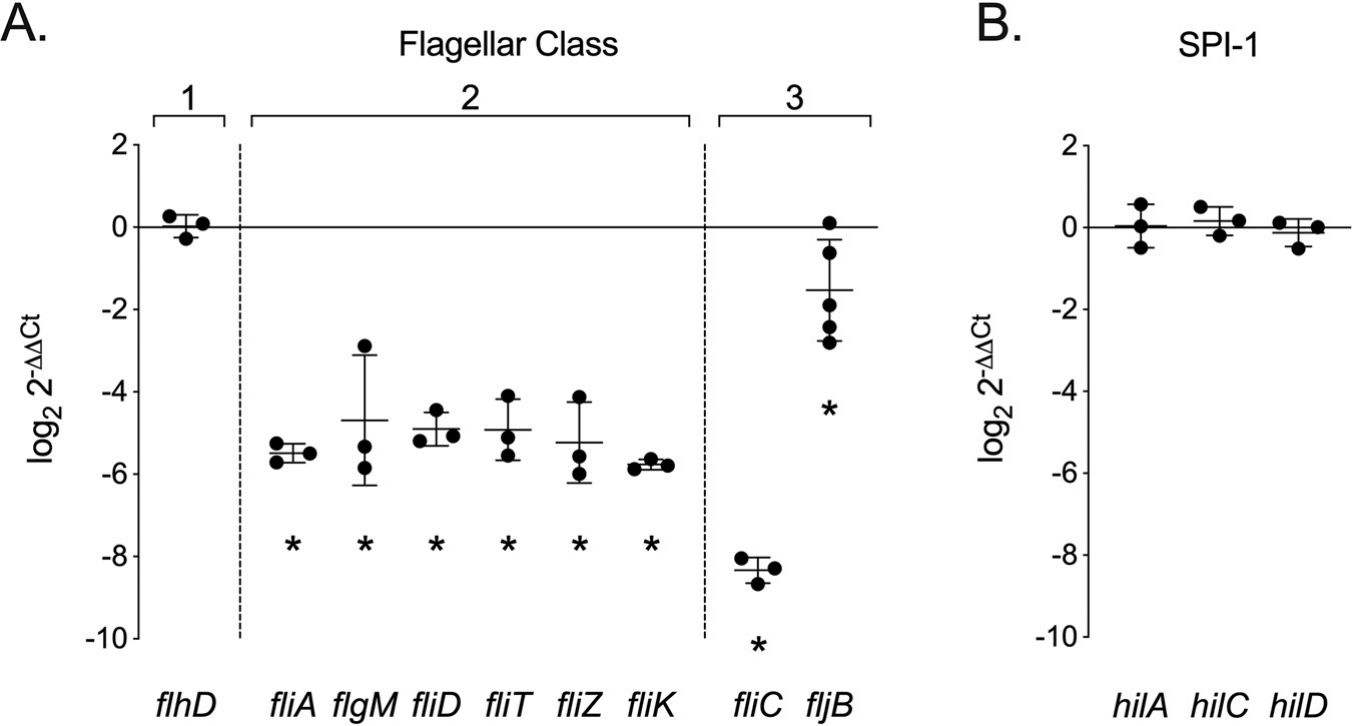
Flagellar class 2 and 3 genes are downregulated in the *ΔyeiE* mutant. Relative expression of the indicated genes from the Δ*yeiE* mutant compared with that of the WT in late exponential growth in rich medium. Each data point represents the mean from triplicate samples from one biological replicate; means ± standard deviations (SD) are indicated. Statistical significance was determined by Student's *t* test (*P* < 0.05). * indicates a significant difference in relative levels of expression of the given gene.

Although the E. coli YeiE regulon does not include genes responsible for flagellar biogenesis (30), our data demonstrate a role for *yeiE* in *S.* Typhimurium flagellum-mediated motility. We hypothesized that YeiE influences the expression of flagellar genes. To investigate the level at which YeiE affects the transcriptional hierarchy of flagellar biogenesis, we measured the mRNA expression of a subset of genes from each of the three flagellar regulatory classes in the WT and Δ*yeiE* mutant. Expression of the class 1 gene *flhD* was not affected by deletion of *yeiE* (Fig. 4A). In contrast, expression of genes from both class 2 and class 3 was significantly downregulated in the *ΔyeiE* mutant (Fig. 4A).

There is regulatory cross talk between the expression of flagellar genes and those borne in Salmonella pathogenicity island 1 (SPI-1), encoding type 3 secretion system 1 and effector proteins needed for the invasion of epithelial cells (38–40). To rule out an effect of *yeiE* on SPI-1 gene expression, we measured the mRNA expression of three SPI-1 regulators in the WT and Δ*yeiE* mutant. We observed no difference in expression of *hilA*, *hilC*, or *hilD* in the Δ*yeiE* mutant (Fig. 4B), suggesting that *yeiE* does not impact SPI-1 gene expression. These data are consistent with our prior work demonstrating no role for *yeiE* in the activation of a promoter for a type 3 secretion system 1 apparatus protein (24). These findings suggest that YeiE promotes the expression of flagellar class 2 genes through an SPI-1-independent process.

### Characterization of the Δ*yeiE* suppressor mutant.

**FIG 5:**
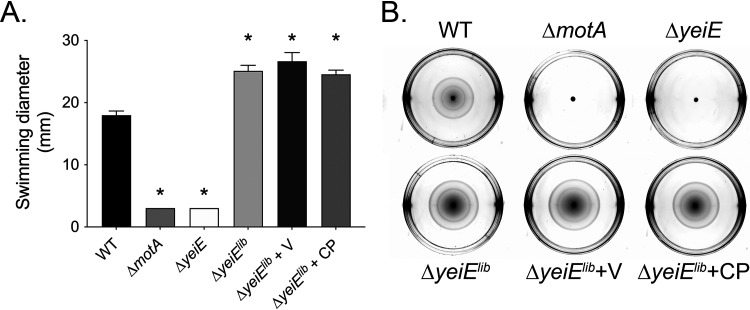
The *ΔyeiE*^lib^ mutant is hypermotile. Normalized overnight cultures of the WT (HA420), *ΔmotA* mutant (JE1202), *ΔyeiE* mutant (JE973), *ΔyeiE*^lib^ mutant (JE1681), *ΔyeiE*^lib^ mutant with the empty vector (Δ*yeiE*^lib^ *+* V; JE2014), and complemented Δ*yeiE*^lib^ mutant (Δ*yeiE*^lib^ *+* CP; JE2016) were spotted onto swimming plates. Statistical significance was determined by Student's *t* test (*P* < 0.05). * indicates a significant difference between the WT and mutant. (B) Representative photographs of swimming plates from one experiment.

In prior work evaluating the role of *S.* Typhimurium genes in the stimulation of the neutrophil respiratory burst, we found that the effects of the Δ*yeiE* mutation in a strain obtained from the kanamycin-resistant Salmonella Typhimurium ATCC 14028s single-gene-deletion library (Δ*yeiE*^lib^ strain) differed from those of the Δ*yeiE* mutation in a clean genetic background (24, 41). To establish whether the differential effects were due to a motility difference between the Δ*yeiE* mutants, we measured the motility of the two Δ*yeiE* mutants on semisolid agar. The Δ*yeiE* mutant in the clean genetic background was amotile, whereas the *ΔyeiE*^lib^ mutant was hypermotile compared to the WT organism (Fig. 5). Furthermore, the complemented Δ*yeiE*^lib^ mutant remained hypermotile (Fig. 5), suggesting that the hypermotility is not due to disruption of *yeiE* in the Δ*yeiE*^lib^ mutant. Therefore, we hypothesized that a suppressor mutation was the likely cause of the observed hypermotility of the Δ*yeiE*^lib^ mutant.

We performed whole-genome sequencing of the amotile Δ*yeiE* mutant and the hypermotile Δ*yeiE*^lib^ suppressor mutant to establish the identity of the suppressor mutation. In a comparison with the published ATCC 14028s genome sequence (42), we identified three single-nucleotide variants (SNVs) in the amotile Δ*yeiE* mutant and three SNVs in the Δ*yeiE*^lib^ suppressor mutant that were different between the two genomes (see Tables S1 and S2 in the supplemental material). In the amotile mutant, we found an SNV in *gyrA* (260T>C) which confers nalidixic acid resistance and is consistent with the phenotypic nalidixic acid resistance in the amotile Δ*yeiE* mutant (Table 1). We also identified an SNV in *eaeH* on the 94-kb virulence plasmid and a single-nucleotide deletion in *cyoB* leading to a frameshift resulting in a predicted truncation of the protein from 664 amino acids to 278 amino acids. CyoB encodes cytochrome *bo* ubiquinol oxidase subunit I, which helps generate the proton-motive force (43). Since the proton-motive force drives flagellum-mediated motility, the predicted CyoB truncation may play a contributing role in its decreased motility (44). However, complementation in *trans* reverses the Δ*yeiE* mutant motility defect (Fig. 1), suggesting that CyoB truncation is not the likely cause of the motility defect.

**TABLE 1:** Bacterial strains and plasmids

Strain or plasmid	Genotype or description	Reference or source
Strains		
HA420	ATCC 14028.s (spontaneous Nal^r^)	59
JE973	HA420 Δ*yeiE*::*kan* (Nal^r^ Kan^r^)	24
JE1202	HA420 Δ*motA*::*kan* (Nal^r^ Kan^r^)	35
JE1511	JE973 carrying pWSK29 (Nal^r^ Kan^r^ Amp^r^)	This study
JE1513	JE973 carrying pWSK29::*yeiE* (Nal^r^ Kan^r^ Amp^r^)	This study
JE1681	14028.s Δ*yeiE*::*kan* (Kan^r^)	41
JE1389	HA420 Δ*yeiE*::*frt* (Nal^r^)	This study
JE1699	HA420 Δ*STM1697*::*cm* (Nal^r^ Cm^r^)	This study
JE1907	JE973 Δ*STM1697*::*cm* (Nal^r^ Kan^r^ Cm^r^)	This study
JE1915	HA420 Δ*fliC*::*kan* Δ*fljB*::*cm* (Nal^r^ Kan^r^ Cm^r^)	This study
JE1919	HA420 Δ*fliC*::*frt ΔfljB*::*frt* (Nal^r^)	This study
JE1921	HA420 Δ*fliC*::*frt ΔfljB*::*frt ΔyeiE*::*kan* (Nal^r^ Kan^r^)	This study
JE2014	JE1681 carrying pWSK29 (Kan^r^ Amp^r^)	This study
JE2016	JE1681 carrying pWSK29::*yeiE* (Kan^r^ Amp^r^)	This study

Plasmids		
pCP20	*flp* recombinase, Amp^r^	51
pWSK29	Cloning vector, Amp^r^	53
pWSK29::*yeiE*	pWSK29::*yeiE* Amp^r^	This study

10.1128/mBio.03680-20.1 TABLE S1 Genome sequencing variants. Variants are differences between the Δ*yeiE* mutants and the ATCC 14028s genome (GenBank accession no. CP001363, chromosome; accession no. CP001362, plasmid). Bold variants are those with reads in both strands (forward/reverse balance of >0.05). Variants in red are those that were identified in both the amotile and suppressor Δ*yeiE* mutants. Download Table S1, XLSX file, 0.01 MB. Copyright © 2021 Westerman et al. 2021 Westerman et al. https://creativecommons.org/licenses/by/4.0/ This content is distributed under the terms of the Creative Commons Attribution 4.0 International license.

10.1128/mBio.03680-20.2 TABLE S2 Characterization of single-nucleotide variants in the Δ*yeiE* and Δ*yeiE*^lib^ mutants identified by whole-genome sequencing. The impact of SNV on the target amino acid sequence of 6 genes was established. *, variants were supported by reads in both strands with over 50% frequency. Download Table S2, XLSX file, 0.01 MB. Copyright © 2021 Westerman et al. 2021 Westerman et al. https://creativecommons.org/licenses/by/4.0/ This content is distributed under the terms of the Creative Commons Attribution 4.0 International license.

Three SNVs were identified in the *ΔyeiE*^lib^ suppressor mutant (Tables S1 and S2). An SNV identified in *hrpA* (248G>T) resulted in a threonine-to-asparagine amino acid sequence change that is unlikely to alter protein function due to replacement of one polar amino acid for another. Two other SNVs in the *ΔyeiE*^lib^ mutant were in *hupA* and *STM1697.* Published work demonstrated that deletion of *hupA* causes an ∼10% motility reduction compared with the motility of its wild-type parent (45). The *STM1697* mutation (565G>A) resulted in a stop codon after amino acid 188, causing early termination of the protein. Deletion of *STM1697* caused hypermotility (19). Since the *ΔyeiE*^lib^ mutant is hypermotile, the SNV in *STM1697* was considered the most likely cause of the observed phenotype.

### The association between YeiE and STM1697.

**FIG 6:**
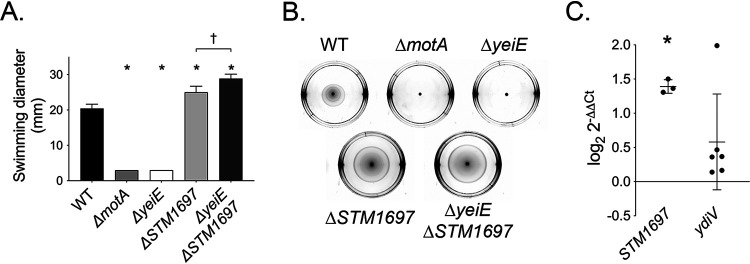
YeiE interacts with STM1697 to alter cell motility. (A) Normalized overnight cultures of the WT (HA420), *ΔmotA* mutant (JE1202), *ΔyeiE* mutant (JE973), *ΔSTM1697* mutant (JE1699), and Δ*yeiE ΔSTM1697* mutant (JE1907) were spotted onto swimming plates. Data analysis was as described in the legend of Fig. 1. (B) Representative photographs of swimming plates from one experiment. (C) Gene expression from the Δ*yeiE* mutant compared with that of the WT as described in the legend of Fig. 4. Each data point represents the mean from triplicate samples from one biological replicate; means ± SD are indicated.

STM1697 inhibits cell motility by preventing RNA polymerase recruitment to FlhD_4_C_2_, leading to repressed flagellar class 2 and 3 gene expression (20). Similarly, flagellar class 2 and 3 genes are downregulated by deletion of *yeiE* without an effect on class 1 gene expression (Fig. 4). Therefore, we hypothesized that there was an interaction between *STM1697* and *yeiE*. We found that the Δ*STM1697* mutant is hypermotile on semisolid agar (Fig. 6A and B), consistent with published reports (19). Deletion of *STM1697* exhibited a dominant effect on the Δ*yeiE* mutant, resulting in the hypermotility of the *ΔyeiE ΔSTM1697* mutant (Fig. 6A and B). These data are similar to our observations of hypermotility for the Δ*yeiE*^lib^ suppressor mutant and suggest that YeiE may repress the expression of the STM1697 FlhD_4_C_2_ repressor.

**FIG 7:**
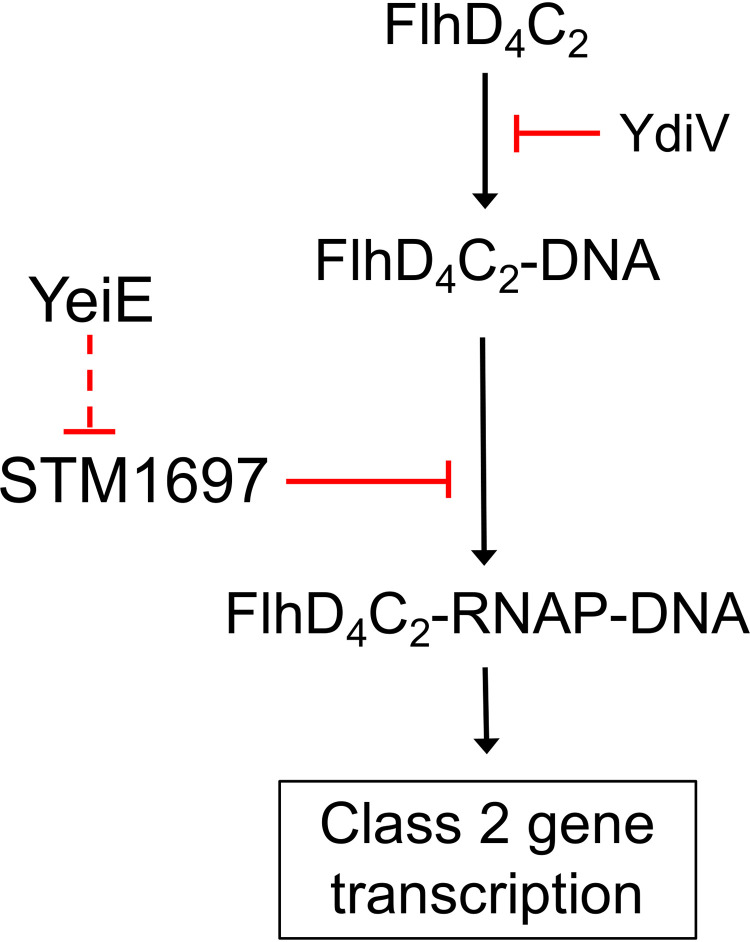
Proposed mechanism for YeiE regulation of cell motility. YeiE acts as a repressor of *STM1697* to promote flagellum-mediated motility. The dotted line indicates an unknown mechanism of repression. RNAP, RNA polymerase.

To test whether YeiE influences the expression of *STM1697*, we determined the relative expression of *STM1697* in the WT and the *ΔyeiE* mutant. We found that the relative expression of *STM1697* is upregulated in the *ΔyeiE* mutant (Fig. 6C). Since YdiV is a related EAL domain-containing protein which also inhibits FlhD_4_C_2_ function, we measured the effects of deletion of *yeiE* on the expression of *ydiV*. We found no significant alteration from *ydiV* expression in the Δ*yeiE* mutant under the conditions tested (Fig. 6C). These data demonstrate that YeiE regulates motility by repressing the expression of the FlhD_4_C_2_ functional repressor *STM1697* (Fig. 7).

## DISCUSSION

The putative LysR-type transcriptional regulator YeiE is critical for Salmonella Typhimurium flagellum-mediated motility and gut colonization. We demonstrate that flagellar class 2 gene expression is reduced in a Δ*yeiE* mutant. Furthermore, we link the positive effect of YeiE on cell motility with STM1697, an anti-FlhD_4_C_2_ factor, and propose that YeiE is a repressor of *STM1697* (Fig. 7).

E. coli and *S.* Typhimurium share a core genome, with approximately 70% of their genetic material conserved between the two species (29). *S.* Typhimurium YeiE (STM2201/STM14_2717) has 89% amino acid identity and 92% similarity with its E. coli homolog, suggesting high functional similarity (46). YeiE is both an activator and a repressor of transcription, binds DNA upstream and downstream of the promoter, and can occupy the same sequence as RNA polymerase (30). YeiE has more than 100 predicted binding sites in the E. coli MG1655 genome, with target genes enriched in functional groups including energy production, amino acid and inorganic ion transport and metabolism, and iron transport (30). The E. coli *yeiE* regulon also includes numerous genes with roles in the regulation of transcription and translation and genes with unknown or poorly characterized functions (30). There were no genes with a direct relationship to flagellar biogenesis in the E. coli YeiE regulon and no motility defects in a Δ*yeiE* mutant (30, 31). Unlike in E. coli, the *S.* Typhimurium Δ*yeiE* mutant has a severe motility defect that we linked to dysregulation of the FlhD_4_C_2_ inhibitor *STM1697*. STM1697 restricts bacterial motility and adds a layer of flagellar regulation that promotes Salmonella intracellular survival and evasion of the host immune system (20). There is no ortholog of *STM1697* in the E. coli MG1655 genome (19) or in the genomes of several thousand other E. coli and *Shigella* strains (data not shown), explaining the different phenotypes observed for Δ*yeiE* mutants of *S.* Typhimurium and E. coli. There are multiple different mechanisms by which YeiE may influence *STM1697* expression. YeiE may directly repress *STM1697* expression by binding to its promoter to inhibit transcription. YeiE may indirectly influence *STM1697* expression by regulating the expression of an activator or inhibitor of STM1697 transcription. Further work is needed to establish the full YeiE regulon of *S*. Typhimurium and to establish how YeiE represses *STM1697* expression in *S.* Typhimurium.

We observed a significant gut colonization defect for the *S.* Typhimurium Δ*yeiE* mutant that we linked to defective motility. Since YeiE is likely to have numerous regulatory targets within the *S.* Typhimurium genome, it is possible that YeiE also regulates other genes that influence *S.* Typhimurium interactions with the host. For example, *yeiE* responds to DNA damage in E. coli, suggesting that it may be activated during exposure to oxidative and nitrosative stresses encountered by *S*. Typhimurium during infection (47). Furthermore, iron import genes are included in the E. coli *yeiE* regulon, suggesting a potential role for *yeiE* in iron homeostasis during *S.* Typhimurium host colonization (30). Although there are numerous potential mechanisms by which *yeiE* may influence pathogenesis, elimination of the flagellar filament restored virulence to the Δ*yeiE* mutant, suggesting that the regulation of flagellum-mediated motility is the primary YeiE target facilitating *S*. Typhimurium colonization during acute enterocolitis. To our knowledge, this is the first demonstration of a role for YeiE in *S.* Typhimurium pathogenesis.

The related organisms Yersinia enterocolitica and *Cronobacter sakazaki* each encode homologs of both *yeiE* and *STM1697*. The Cronobacter sakazakii *yeiE* homolog (*gpESA_01081*) shares 84% amino acid identity with *S.* Typhimurium YeiE and is required for enterocyte invasion, biofilm formation, neutrophil recruitment, and virulence in neonatal rats (32, 48). The Yersinia enterocolitica *yeiE* homolog (*rscR*) shares 75% identity with *S.* Typhimurium YeiE and limits systemic dissemination of the organism (48, 49). The effects of the *C. sakazaki* and Y. enterocolitica *yeiE* homologs (*gpESA_01081* and *rscR*, respectively) on motility have not yet been characterized. We hypothesize that the role of the *yeiE* homologs in virulence is due, in part, to regulation of the *STM1697* homolog in each of these organisms.

Evaluation of the *ΔyeiE*^lib^ suppressor mutant led to the identification of *STM1697* as a target for YeiE motility regulation. STM1697 is an anti-FlhD_4_C_2_ factor that represses cell motility by binding FlhD, thereby preventing RNA polymerase recruitment to the FlhD_4_C_2_-DNA complex (20). The C terminus of STM1697 is critical for its function, as truncation of the 235-amino-acid protein at residue 192 results in a nonfunctional protein and hypermotility (19). Although the crystal structure of the STM1697-FlhD complex suggests that Tyr180 is the residue closest to the C terminus required for stable STM1697-FlhD interaction (20), our data demonstrate an SNV in *STM1697* causing a stop codon after amino acid 188, and deletion of *STM1697* resulted in the same hypermotile phenotype, confirming prior reports that the C terminus is critical for STM1697 function (see Table S2 in the supplemental material). These data, in combination with prior work (19), suggest that one or more residues located even closer to the C terminus may be required for STM1697 stability or function *in vivo*.

Salmonella uses flagellum-mediated motility to interact with host cells, driving successful gut colonization; however, it is critical for Salmonella to repress flagellin expression when residing within host cells to evade immune detection (2, 4, 7, 50). Our data suggest that YeiE functions as a regulatory control point to promote flagellar motility by inhibiting the anti-FlhD_4_C_2_ factor STM1697. The promotility effect of STM1697 inhibition by YeiE likely facilitates initial gut colonization, whereas STM1697-mediated repression of flagellin aids immune evasion once Salmonella is located intracellularly (20). The regulatory functions of LysR regulators are influenced by small-molecule coinducers that sense environmental conditions (25, 26). Further molecular characterization of YeiE and the identification of its coinducer will help to delineate how this LysR regulator exerts control over cell motility during infection.

We definitively link *yeiE* with *S.* Typhimurium motility and demonstrate its requirement for gut colonization using two animal models of enterocolitis. We propose that YeiE serves as a control point for flagellar regulation through inhibition of the anti-FlhD_4_C_2_ factor STM1697, although additional work is needed to establish the mechanism by which YeiE influences *STM1697* expression. Tight control of flagellar biogenesis is critical to facilitate pathogenesis. Whereas the flagellum-based motility state is needed for gut colonization, elimination of flagellins is critical to evade immune detection. Our work demonstrates that YeiE serves as a key regulatory control point for flagellar biogenesis to facilitate *S.* Typhimurium enteropathogenesis.

## MATERIALS AND METHODS

### Bacterial strains and growth conditions.

All bacterial strains are derivatives of Salmonella enterica serotype Typhimurium ATCC 14028s (Table 1). Mutations were moved into a clean genetic background by P22 transduction, and antibiotic cassettes were removed by *flp*-mediated recombination as previously described (51, 52). Bacteria were grown on Luria-Bertani (LB)-Miller agar or in LB broth at 37°C with agitation (225 rpm) unless otherwise noted. Medium was supplemented with the following antibiotics, as appropriate: nalidixic acid (50 mg/liter), chloramphenicol (20 mg/liter), kanamycin (50 mg/liter), and carbenicillin (100 mg/liter).

### Complementing plasmid construction.

Genomic DNA was isolated from the wild-type organism using the GenElute bacterial genomic DNA kit (Sigma-Aldrich). A 1.5-kb PCR product of *yeiE*, including its native promoter, was generated from genomic DNA by PCR using Q5 polymerase (New England Biolabs), with an annealing temperature of 72°C and an extension time of 40s for 30 cycles. Restriction sites for endonucleases were incorporated into the following primers to facilitate cloning: STM2201BamH1Fwd, 5′-GTCGGATCCTGCCTGTCCAGACCAAAGA-3′, and STM2201Kpn1Rev, 5′-GTCGGTACCTGCGCGGTTATAAGAGACCT-3′. The expected size of the PCR product was confirmed by agarose gel electrophoresis. The PCR product was digested with restriction endonucleases BamHI and KpnI (New England Biolabs) and purified with the QIAquick PCR purification kit (Qiagen). The insert was cloned into the pWSK29 vector, sequentially digested with BamHI and KpnI (53). Ligation was performed overnight at 14°C with T4 DNA ligase (New England Biolabs). The resulting construct was transformed into DH5α Escherichia coli by heat shock. Transformants were obtained by selection on LB agar with carbenicillin and X-gal (5-bromo-4-chloro-3-indolyl-β-d-galactopyranoside; 40 μg/ml). Plasmids were isolated (QIAprep miniprep kit; Qiagen), and the correct insert size was confirmed by agarose gel electrophoresis of digested plasmids. The insert sequence was confirmed by Sanger sequencing (Eton Bioscience). The complementing plasmid (CP) was transformed into restriction-deficient modification-positive *S.* Typhimurium LB5000 by electroporation, and transformants were isolated by selection on LB agar with carbenicillin (54). The complementing plasmid and empty vector were isolated and then transformed into the indicated mutants by electroporation. The resulting strains were purified by streaking them twice for single colonies on LB with carbenicillin and stored in glycerol stocks at −80°C.

### Growth curves.

Overnight cultures were diluted 1:100 into 50 ml of LB or M9 minimal medium and grown at 37°C with agitation (225 rpm) for 24 h. M9 minimal medium (48 mM Na_2_HPO_4_, 22 mM KH_2_PO_4_, 9 mM NaCl, 19 mM NH_4_Cl, 0.1 mM CaCl_2_, and 2 mM MgSO_4_) was supplemented with 0.2% (wt/vol) dextrose as a carbon source (55). Samples were taken hourly for 6 h and once at 24 h, serially diluted, and plated for enumeration of CFU per milliliter.

### Bacterial motility assays.

Swimming assays were performed on semisolid agar as previously described (56). Swimming motility was assayed on plates containing 0.3% Difco Bacto agar (LB-Miller base, 25 g/liter). Overnight cultures were grown at 37°C with agitation, and the cell concentration was normalized by optical density. Bacterial strains were spotted (3 μl) onto swimming plates and incubated at 37°C. The widest diameter of each colony was measured in two intersecting planes after 4 h of incubation. Images of swimming plates were obtained on ChemiDoc MP (Bio-Rad) after 5 h of incubation. Each assay was performed in triplicate on three independent occasions.

### Calf infections.

Calf infections were approved by the North Carolina State University Institutional Animal Care and Use Committee (protocol 15-047-B). Calves were obtained from the university teaching herd and transferred to individual AALAC-approved housing within 8 h following birth. Calves were administered a commercial colostrum replacer (AgriLabs Colostrx CR) by an esophageal feeder upon arrival and 2 h following initial administration. Calves were fed milk replacer twice daily and provided hay and water *ad libitum*. At 1 day of age, adequate passive transfer of immunity was estimated by measuring serum total solids with a refractometer. Fecal cultures were performed at least twice weekly using selective media to ensure that calves were not shedding Salmonella prior to experimental infection (57).

In preparation for the ligated ileal loop surgery, bacteria were grown overnight at 37°C with agitation in LB broth. Overnight cultures were subcultured 1:100 into LB broth and incubated for 3 to 4 h at 37°C with agitation. Bacteria were washed twice in sterile LB broth, and the mutant and WT were mixed in a 1:1 ratio based on optical density (600 nm). Loops were inoculated with approximately 10^9^ CFU of the mixture in 3 ml LB broth.

Ligated ileal loop surgery was performed on 5 bull calves (3 Jersey and 2 Holstein) aged 3 to 6 weeks, as previously described (57). At 12 h postinfection, the luminal contents, mucus overlying the epithelium, and epithelial tissue were harvested, collected in phosphate-buffered saline (PBS), homogenized, serially diluted, and plated to determine numbers of CFU. The competitive index (CI) between the WT and mutant was determined as the ratio of WT to mutant bacteria after infection normalized to the ratio in the inoculum.

### Mouse infections.

Mouse infections were approved by the University of Wisconsin—Madison Institutional Animal Care and Use Committee (protocol no. V006255). The acute murine colitis model was used as previously described, with 10- to 12-week-old female C57BL/6J mice obtained from Jackson Laboratories (strain 000664) (37). Mice were administered 20 mg streptomycin in sterile water by oral gavage 24 h prior to infection. Overnight bacterial cultures were washed in PBS, and mice were infected with approximately 10^8^ CFU of a 1:1 mixture of the two competing strains by oral gavage. Mice were euthanized 72 h postinfection. Organs were harvested, homogenized, serially diluted, and plated for enumeration of CFU. The competitive index was determined as for calf infections.

### Genome sequencing and analysis.

Genomic DNA from the amotile (JE973) and hypermotile (JE1681) *ΔyeiE* mutants was isolated (GenElute bacterial genomic DNA kit; Sigma-Aldrich) and submitted to the North Carolina State University Genomic Sciences Laboratory. Genomic DNA (gDNA) quality was analyzed using TapeStation (Agilent), and library preparation and whole-genome shotgun sequencing was performed using a MiSeq platform (PE300; Illumina). Raw sequencing data were provided as demultiplexed .fastq files. Variants were found using the CLC Genomics Workbench (Qiagen) resequencing analysis module. Reads were first mapped to the published ATCC 14028s genome (42) (NCBI accession no. NC_016856.1), followed by local realignment. Variants were then detected using the fixed-ploidy variant detection tool. Variants that were supported by reads in both strands with more than 50% frequency were further investigated.

### Gene expression analyses.

Overnight cultures of the WT and Δ*yeiE* mutant were diluted 1:100 into LB broth and grown at 37°C with agitation for 3.5 h. Total RNA was isolated using TRIzol (Invitrogen) according to the manufacturer’s instructions. RNA quantity and integrity were determined using a Qubit 4 fluorometer (Invitrogen), and samples with an RNA IQ value of >7.5 (Qubit RNA IQ assay; Invitrogen) were considered of good quality and were used for downstream applications. Removal of contaminating DNA was performed using TURBO DNase (Invitrogen) according to the manufacturer’s instructions.

Reverse transcription (RT) of RNA to cDNA was performed by random-hexamer-dependent amplification using TaqMan reverse transcription reagents according to the manufacturer’s instructions (Invitrogen). Real-time PCR was performed using probe-based 5′ nuclease assays (IDT PrimeTime quantitative PCR [qPCR]). Primer and probe sets (see Table S3 in the supplemental material) were designed using the IDT PrimerQuest tool (https://www.idtdna.com/SciTools) and incorporated 6-carboxyfluorescein (FAM)/ZEN^TM^ (IDT trademarked dark quencher)/Iowa black fluorescent quencher (IBFQ) doubly quenched probes (Integrated DNA Technologies). Cycling parameters were an initial 95°C polymerase activation step for 2 min, followed by 40 cycles of 95°C for 15 s and 60°C for 1 min, performed on a StepOnePlus real-time PCR system (Applied Biosystems). Amplification efficiency was determined to be 90 to 100% for all primer/probe sets (Table S4). All qPCR products were confirmed to have a single product of the expected size by agarose gel electrophoresis. All real-time PCR assays were performed in three technical repeats using cDNA from at least 3 biological replicates. The change in threshold cycle (Δ*C_t_*) for each gene of interest was determined for each strain, using *rpoD* as the reference gene for normalization (58). The relative expression of each gene of interest was determined by comparing the Δ*C_t_* of the mutant to that of the WT using the comparative threshold (ΔΔ*C_t_*) method. All quantitative RT-PCR data are available in Table S4.

10.1128/mBio.03680-20.3 TABLE S3 Primer and probe sequences for qRT-PCR. Download Table S3, XLSX file, 0.01 MB. Copyright © 2021 Westerman et al. 2021 Westerman et al. https://creativecommons.org/licenses/by/4.0/ This content is distributed under the terms of the Creative Commons Attribution 4.0 International license.

10.1128/mBio.03680-20.4 TABLE S4 qRT-PCR primer efficiencies and raw *C_t_* data. Download Table S4, XLSX file, 0.06 MB. Copyright © 2021 Westerman et al. 2021 Westerman et al. https://creativecommons.org/licenses/by/4.0/ This content is distributed under the terms of the Creative Commons Attribution 4.0 International license.

### Data analysis.

Statistical significance was determined by a two-tailed Student *t* test, with significance set at a *P *of <0.05. Analyses were performed using GraphPad Prism version 8.0.

### Data availability.

The raw DNA sequences were deposited in GenBank (BioProject accession no. PRJNA704982).
